# Dynamic Enhancement Pattern on CT for Predicting Pancreatic Neuroendocrine Neoplasms with Low PAX6 Expression: A Retrospective Observational Study

**DOI:** 10.3390/diagnostics10110919

**Published:** 2020-11-09

**Authors:** Koichiro Kimura, Junichi Tsuchiya, Yoshio Kitazume, Mitsuhiro Kishino, Keiichi Akahoshi, Atsushi Kudo, Shinji Tanaka, Minoru Tanabe, Ukihide Tateishi

**Affiliations:** 1 Department of Diagnostic Radiology, Graduate School of Medicine, Tokyo Medical and Dental University, 1-5-45 Yushima, Bunkyo-ku, Tokyo 1138510, Japan; tcymrad@tmd.ac.jp (J.T.); ktzmmrad@tmd.ac.jp (Y.K.); ksnmrad@tmd.ac.jp (M.K.); ttisdrnm@tmd.ac.jp (U.T.); 2 Department of Hepatobiliary and Pancreatic Surgery, Graduate School of Medicine, Tokyo Medical and Dental University, 1-5-45 Yushima, Bunkyo-ku, Tokyo 1138510, Japan; akahmsrg@tmd.ac.jp (K.A.); kudomsrg@tmd.ac.jp (A.K.); tana.msrg@tmd.ac.jp (M.T.); 3 Department of Molecular Oncology, Graduate School of Medicine, Tokyo Medical and Dental University, 1-5-45 Yushima, Bunkyo-ku, Tokyo 1138510, Japan; tanaka.monc@tmd.ac.jp

**Keywords:** pancreas, neuroendocrine Tumors, PAX6

## Abstract

Paired box 6 (PAX6) is a transcription factor that plays a critical role in tumor suppression, implying that the downregulation of PAX6 promotes tumor growth and invasiveness. This study aimed to examine dynamic computed tomography (CT) features for predicting pancreatic neuroendocrine neoplasms (Pan-NENs) with low PAX6 expression. We retrospectively evaluated 51 patients with Pan-NENs without synchronous liver metastasis to assess the pathological expression of PAX6. Two radiologists analyzed preoperative dynamic CT images to determine morphological features and enhancement patterns. We compared the CT findings between low and high PAX6 expression groups. Pathological analysis identified 11 and 40 patients with low and high PAX6 expression, respectively. Iso- or hypoenhancement types in the arterial and portal phases were significantly associated with low PAX6 expression (*p* = 0.009; *p* = 0.001, respectively). Low PAX6 Pan-NENs showed a lower portal enhancement ratio than high PAX6 Pan-NENs (*p* = 0.044). The combination based on enhancement types (iso- or hypoenhancement during arterial and portal phases) and portal enhancement ratio (≤1.22) had 54.5% sensitivity, 92.5% specificity, and 84.3% accuracy in identifying low PAX6 Pan-NENs. Dynamic CT features, including iso- or hypoenhancement types in the arterial and portal phases and lower portal enhancement ratio may help predict Pan-NENs with low PAX6 expression.

## 1. Introduction

Pancreatic neuroendocrine neoplasms (Pan-NENs) are a heterogeneous group of epithelial tumors that originate from the neuroendocrine cells of the pancreas [1]. Pan-NENs are rare, with an annual prevalence of only <1 per 100,000 persons. However, their incidence is gradually increasing [1,2]. According to the 2017 World Health Organization (WHO) classification, Pan-NENs are divided into well-differentiated tumors (neuroendocrine tumor [NET]) and poorly differentiated tumors (neuroendocrine carcinoma [NEC]). NET is further subdivided into grades 1 (G1), 2 (G2), and 3(G3) according to the malignant potential determined by Ki-67 proliferative index and/or mitotic count [3]. All Pan-NENs have malignant potential, and the five-year survival rate is <43% [4]. Aggressive cases show extrapancreatic invasion and metastases with poor prognosis. Notably, the liver is the most common site for metastasis of Pan-NENs [5], and patients with hepatic metastases show poorer survival rate than those without hepatic metastases [6,7].

Paired box 6 (PAX6) is a transcription factor that is tightly linked with normal organ development and belongs to the paired box family [8]. It is crucial in the embryonic development of several organs, including the central nervous system, eyes and pancreas [9]. More specifically, basic research demonstrated that PAX6 is required for normal vasculature development in the telencephalon and choroid [10,11]. This transcription factor plays a critical role in the correct differentiation and maintenance of function in the islets of Langerhans [12,13,14,15], and also in in the prevention of early-onset diabetes [16]. In addition to organ development, PAX6 is also expressed in a wide range of cancer cell lines and helps inhibit the growth of glioblastoma and prostate carcinoma cell lines in vitro [8,17,18,19,20,21], implying that the downregulation of PAX6 promotes tumor growth and invasiveness. In several types of cancer, low PAX6 expression results in a worse prognosis [22,23,24,25]. PAX6 is regarded as an immunohistochemical marker for NET of pancreatic origin [26]. The majority of Pan-NENs appear as immunohistochemically positive for PAX6 staining [27]. Furthermore, intermediate-grade Pan-NENs showed significantly lower PAX6 staining intensity than low-grade Pan-NENs [26]. A previous study from our institution reported that Pan-NENs with downregulated pancreatic beta-cell genes, involving PAX6, developed postoperative metachronous hepatic metastasis and had poor outcomes [22]. Additionally, that study verified that PAX6 could predict the metachronous hepatic metastasis-free survival of patients with postoperative Pan-NENs more accurately than conventional histologic risk factors, included in the 2017 WHO classification such as the Ki-67 proliferative index, mitotic count, and level of tumor differentiation [22]. This previous study found that PAX6 was the new significant prognostic factor for Pan-NENs. The PAX6 expression assessment is based on a sampling from a biopsy or surgery. However, assessing a single random sample may risk an incorrect evaluation for the entire lesion due to the intratumoral heterogeneity. Therefore, a non-invasive prediction technique is also essential in pointing out the possibility of low PAX6 expression.

Dynamic computed tomography (CT) is a useful imaging technique to evaluate the characteristics of Pan-NENs. Typically, Pan-NENs appear as small, well-defined masses with homogeneous hyperenhancement in the arterial phase because of their high vascularization [28,29]. However, Pan-NENs showing arterial iso- or hypoenhancement are not rare [30,31]. Previous studies showed that iso- or hypoenhancement in the arterial phase indicated low vascularization and poor outcome in Pan-NENs [32,33,34,35]. However, no study has investigated the relationship between CT features and subtypes of Pan-NENs according to PAX6 expression. Accordingly, this study aimed to examine preoperative CT features for predicting low expression of PAX6 in Pan-NENs without synchronous liver metastasis.

## 2. Materials and Methods

### 2.1. Patients

This retrospective study was approved by the institutional review board of the Tokyo Medical and Dental University, and the requirement for informed consent was waived (the project identification code: M2018-146, the date of approval: 18 September 2018). We reviewed electronic medical records of 70 patients who had a history of curative surgical treatment and were histologically diagnosed with Pan-NENs without synchronous liver metastasis between April 2000 and March 2018. Fourteen patients were excluded due to lack of available preoperative CT images (*n* = 5), preoperative CT with no DICOM data sets (*n* = 2), multiphasic dynamic CT without both arterial and portal phases (*n* = 7), and no detectable lesions on preoperative CT (*n* = 3). We also excluded patients with multiple lesions because it was difficult to match individual lesions with data of PAX6 expression (*n* = 2). Therefore, a total of 51 patients with Pan-NENs who had no synchronous liver metastases were included in the analysis. They were divided into two groups: low and high PAX6 expression. All patients were followed up until 31 March 2018, and we counted the number of metachronous hepatic metastasis and death that occurred during follow-up.

### 2.2. Pathological Analysis

**Figure 1:**
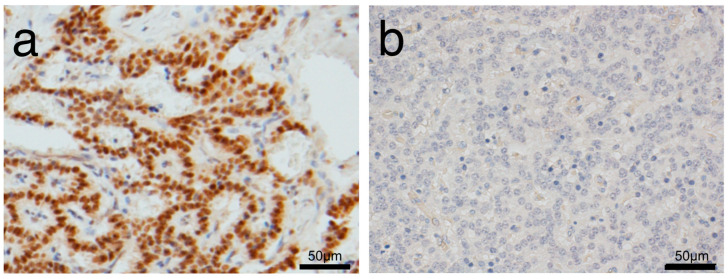
Representative immunohistochemistry images of high PAX6 expression; and (**a**) low PAX6 expression; (**b**) in primary tumors.

The diagnosis of Pan-NENs was confirmed based on histologic findings and immunohistochemical expression of chromogranin A and synaptophysin. Pathological grading was performed according to the 2010 WHO classification. PAX6 expression in resected pancreatic samples from patients was evaluated using immunohistochemical staining. The detailed methods for PAX6 immunohistochemistry were as follows. Formalin-fixed paraffin-embedded tissue samples were sectioned into 4-μm-thick slices. For antigen retrieval, slides were subjected to microwave radiation in 0.01 M citrate buffer solution (pH 6.0) for 15 min and then incubated with an anti-PAX6 antibody (sc-81649, Santa Cruz Biotechnology, Texas, U.S.A.) at a 1:200 dilution for 24 h at 4 °C. The secondary antibody used was Histofine Simple Stain MAX Peroxidase (414144F, AS ONE, Osaka, Japan). The incubation time was 1 h at room temperature. All tissue sections were counterstained with hematoxylin. The criteria for determining low and high PAX6 expression are as follows. PAX6 was expressed in all normal islets of Langerhans, which were considered the internal control for PAX6 expression. Tumor tissues with an equal or higher degree of staining of PAX6 when compared with the islet cells were categorized as high PAX6 expression (Figure 1a). Tumor tissues with a lower degree of staining, when compared with the islet cells, were judged as having low PAX6 expression (Figure 1b). Two observers (A.K. and K.A.) independently evaluated PAX6 expression in the primary tumor in a blinded manner. In case of discrepancies, a consensus was reached by joint review. We also extracted the Ki-67 proliferative index and mitotic count of the primary tumors from the pathology reports. We classified the Ki-67 proliferative index and mitotic count respectively into two groups (≤2%: low Ki-67, >2%: high Ki-67; and <2 n/10 high power field: low miotic, ≥2 n/10 high power field: high miotic) according to 2010 WHO classification.

### 2.3. CT Imaging Techniques and Analysis

Due to this was a retrospective study, 11 (21.6%) patients who had undergone preoperative dynamic CT studies at institutions outside our university hospital were included. Therefore, the CT protocols varied, but all these CT studies included unenhanced scans and arterial and portal phase scans. Overall, 40 (78.4%) patients had undergone preoperative dynamic CT studies at our institution, and again all included unenhanced scans and arterial and portal phase scans. The injection protocol used at our institution is described in Appendix A1.

Two experienced radiologists (J.T. and Y.K.) independently reviewed the preoperative CT images using picture archiving and communication systems, and interobserver agreement was assessed for the CT features. Discrepancies were resolved by a third senior radiologist (M.K.). The radiologists were only aware of the histopathological diagnosis of Pan-NENs, but they were blinded to the pathological grading, Ki-67 proliferative index, mitotic count, and PAX6 expression.

These morphological features of Pan-NENs were analyzed: (1) Ttumor location in the pancreas (head, body, or tail); (2) maximum size; (3) shape (round, oval, or lobulated); (4) margin (well-defined or ill-defined); (5) presence of cystic changes; (6) calcifications; (7) visible intratumoral vessel in the arterial phase; (8) invasion into adjacent vascular structures; (9) main pancreatic duct dilatation (defined as diameter ≥4 mm); and (10) presence of upstream pancreatic atrophy.

**Figure 2:**
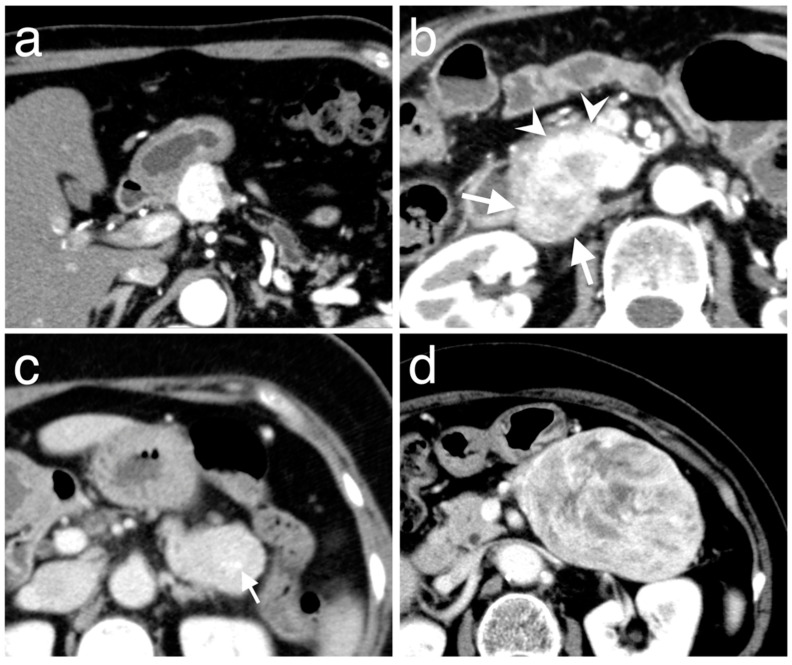
Classification of tumor homogeneity, according to arterial (**a**,**b**) and portal phase (**c**,**d**). (**a**) A 62-year-old man with Pan-NEN shows homogeneous enhancement at the arterial phase over the whole area. (**b**) A 51-year-old woman with Pan-NEN shows heterogeneous enhancement at the arterial phase: the tumor contains a large area of weak to moderate enhancements (arrows) and shows strong enhancement in part of the area (arrowheads). (**c**) A 65-year-old woman with Pan-NEN shows homogeneous enhancement at the portal phase over the whole tumor. The arrow indicates the nodular calcification. (**d**) An 80-year-old woman with Pan-NEN shows heterogeneous enhancement at the portal phase, the tumor contains the area of both strong and weak enhancements.

**Figure 3:**
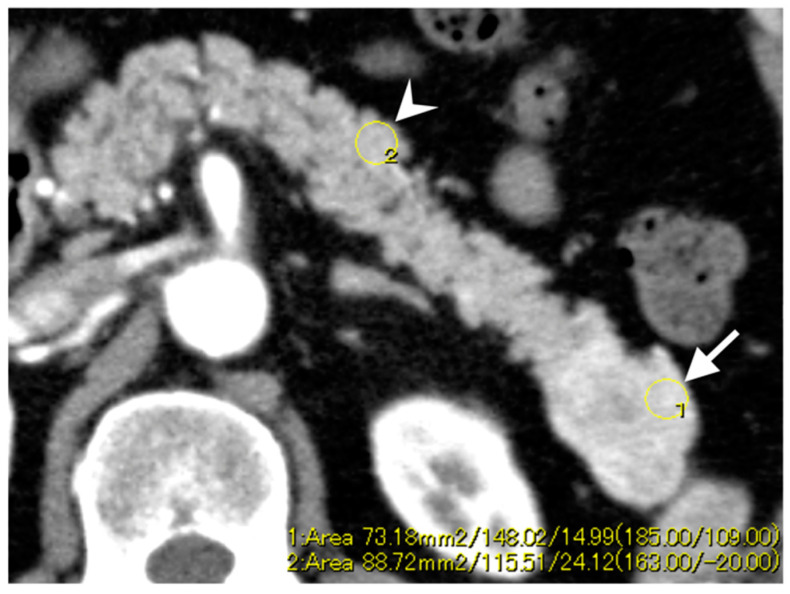
Representative images with region of interest drawn in the Pan-NEN tumor (arrow) and normal pancreatic parenchyma (arrowhead) at the arterial phase.

The enhancement patterns of Pan-NENs in both, the arterial and portal phases were qualitatively assessed. The qualitative evaluation included tumor homogeneity and main enhancement types. Tumor homogeneity was categorized into “homogeneous” (Figure 2a,c) or “heterogeneous” (Figure 2b,d) according to each phase. During each phase, the main enhancement types were visually rated as either “hyperenhancement” or “iso- or hypoenhancement” and depended on comparison with the normal pancreatic parenchyma. A quantitative evaluation was performed as follows. The Hounsfield unit (HU) value was measured by placing the oval region of interest (ROI), which is at least 10 mm^2^, within the tumor and normal pancreatic parenchyma in each of the contrast phase images (Figure 3). If the tumor showed heterogeneous enhancement, the ROI was drawn at the area that showed the main enhancement type. Care was taken to avoid calcifications, cystic changes, adjacent vasculatures, and the main pancreatic duct. The enhancement ratio was defined as the HU value of the primary tumor divided by that of the normal pancreatic parenchyma in the arterial and portal phase.

### 2.4. Statistical Analysis

Cohen’s kappa analysis was used to assess interobserver agreement regarding radiological features. Kappa (k) values were calculated separately for each feature and interpreted as follows: <0.20, slight agreement; 0.21–0.40, fair agreement; 0.41–0.60, moderate agreement; 0.61–0.80, substantial agreement; and 0.81–1.00, almost perfect agreement. We compared the CT features between low and high PAX6 expression groups. Each of the Ki-67 proliferative index and mitotic count groups was also compared to the CT features. Between-group comparisons were performed using Fisher’s exact test for categorical variables. All continuous variables were tested for normality with histogram and the Shapiro-Wilk test. In this study, none of the continuous variables had a normal distribution. Hence, the Mann–Whitney U test was employed for comparing continuous variables between groups. The maximal Youden index, determined using receiver operating characteristic (ROC) analysis, was used to determine the appropriate cut-offs for significant continuous variables. The sensitivity, specificity, accuracy, positive likelihood ratio, and negative likelihood ratio of significant imaging findings and combinations of these features were calculated with 95% confidence intervals (CI). All statistical analyses were performed using EZR version 1.40 (Saitama Medical Center, Jichi Medical University, Tochigi, Japan) and R version 3.6.0 (R Foundation for Statistical Computing, Vienna, Austria). A *p* value less <0.05 was considered statistically significant.

## 3. Results

### 3.1. Comparison of Characteristics between Low and High PAX6 Expression Groups

**Figure 4:**
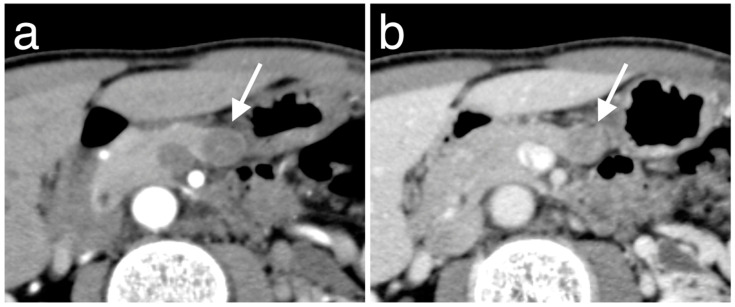
Representative images of Pan-NEN with low PAX6 expression in a 43-year-old man. Compared with normal pancreatic parenchyma, the round mass in the pancreatic tail (arrows) shows hypoenhancement over the entire area in the arterial; and (**a**) portal; (**b**) phases.

**Figure 5:**
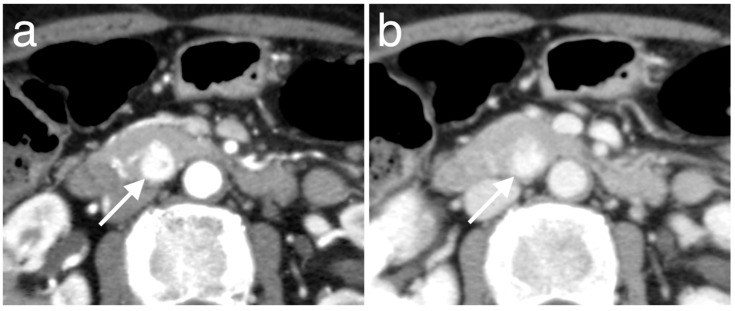
Representative images of Pan-NEN with high PAX6 expression in a 64-year-old woman. Compared with normal pancreatic parenchyma, the round mass in the pancreatic head (arrows) shows a hyperenhancement in the arterial phase; (**a**) persistence of the hyperenhancement in the portal; and (**b**) phase.

**Figure 6:**
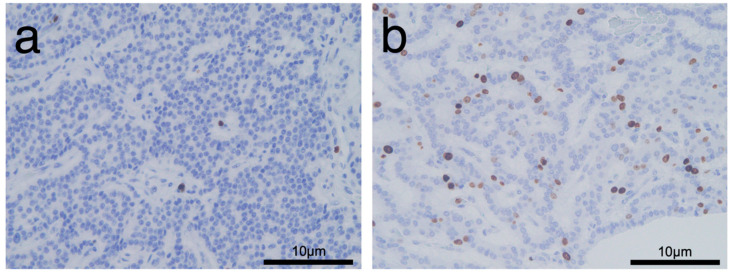
Representative immunohistochemistry images of Ki-67 according to high PAX6 expression (**a**) and low PAX6 expression (**b**) in primary tumors. (**a**) A 57-year-old man with NET G1. Ki-67 immunostaining shows positive in 1.0% of tumor cells. (**b**) A 77-year-old man with NET G2. Ki-67 immunostaining shows positive in 9.4% of tumor cells.

The clinicopathologic characteristics of the 51 patients according to PAX6 expression are shown in Table 1. Overall, 11 (21.6%) patients showed low PAX6 expression (Figure 4a,b), and 40 (78.4%) showed high PAX6 expression (Figure 5a,b). There were 2 patients (3.9%) who were diagnosed with genetic disorder. Of those patients, one who had von Hippel-Lindau disease was classified into the low PAX6 expression group, and the other patient with multiple endocrine neoplasia type 1 syndrome was classified into the high PAX6 expression group. Approximately half the patients with low PAX6 expression and the majority of patients with high PAX6 had NET G1 (*n* = 6/11, 54.5% vs. *n* = 33/40, 82.5%). NET G2 or NEC G3 was more frequent in the low than in the high PAX6 expression group (*n* = 5/11, 45.5% vs. *n* = 7/40, 17.5%). Pathological grading was significantly associated with PAX6 expression (*p* = 0.049). The mean Ki-67 proliferative index was significantly higher for the low than for the high PAX6 expression group (6.25% vs. 1.81%, *p* = 0.021). Figure 6a,b shows representative immunohistochemistry images of Ki-67 according to PAX6 expression. The mean mitotic count of the low PAX6 expression group was higher than that of the high PAX6 expression group (1.45 vs. 0.80, *p* = 0.11). The total rate of metachronous hepatic metastasis during follow-up was 5.9% (*n* = 3/51); 2/11 (18.2%) in the low and 1/40 (2.5%) in the high PAX6 expression group (*p* = 0.114). Two patients died during follow-up, both in the low PAX6 expression group (*n* = 2/11, 18.2% vs. *n* = 0/41, 0%, *p* = 0.043).

### 3.2. Morphological Features of Pan-NENs Associated with Low or High PAX6 Expression

The morphological features of the 51 Pan-NEN patients are summarized in Table 2. Interobserver agreement for the radiological features was fair to almost perfect (k = 0.45 for margin; k = 0.92 for calcifications). The median tumor size was slightly larger in the low than in the high PAX6 expression group (18.6 mm vs. 15.5 mm, *p* = 0.24). Pan-NENs with low PAX6 expression tended to exist in the pancreatic tail (*n* = 6/11, 54.5%, *p* = 0.28) and appeared as an oval mass (*n* = 7/11, 63.6%, *p* = 0.44). Calcifications, invasion into adjacent vascular, and upstream pancreatic atrophy occurred more frequently in the low PAX6 expression group than in the high PAX6 expression group (*n* = 4/11, 36.4% vs. *n* = 4/40, 10.0%, *p* = 0.055; *n* = 2/11, 18.1% vs. *n* = 3/40, 7.5%, *p* = 0.29; *n* = 2/11, 18.1% vs. *n* = 1/40, 2.5%, *p* = 0.11). However, there was no significant difference in morphological features between the low and high PAX6 expression groups.

### 3.3. Enhancement Patterns of Pan-NENs Related to Low or High PAX6 Expression

**Figure 7:**
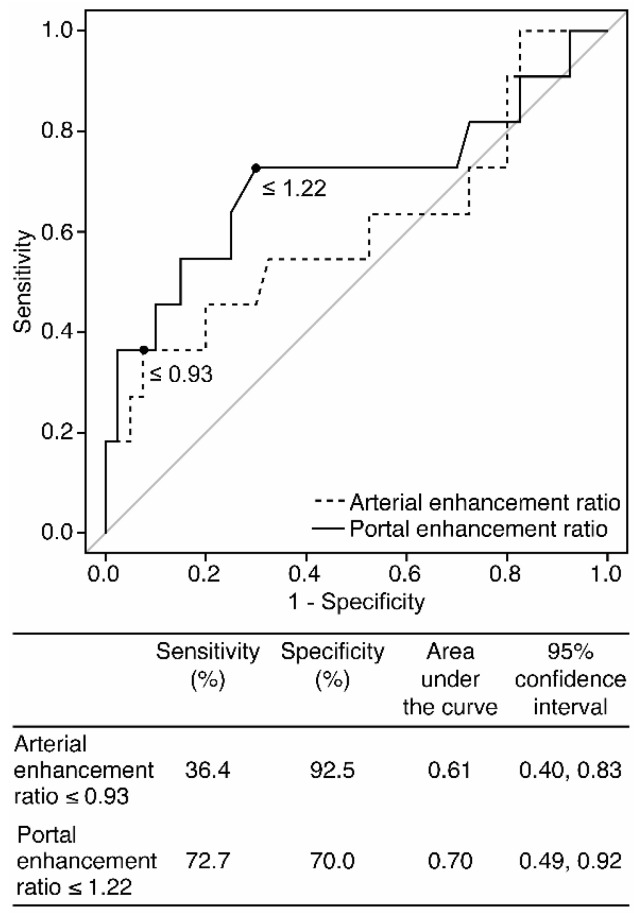
Receiver operating characteristic curves based on arterial and portal enhancement ratio and the optimal cut-off values for differentiating low PAX6 from high PAX6 expression of Pan-NENs.

The tumor homogeneity, main enhancement types, and enhancement ratio according to PAX6 expression are shown in Table 3. Iso- or hypoenhancement types in the arterial phase were significantly more frequent in the low than in the high PAX6 expression group (*n* = 7/11, 63.6% vs. *n* = 8/40, 20.0%, *p* = 0.009). In addition, iso- or hypoenhancement types in the portal phase were significantly more frequent in the low PAX6 than in the high PAX6 expression group (*n* = 8/11 72.7% vs. *n* = 7/40 17.5%, *p* = 0.001). The enhancement ratio in the arterial phase of the low PAX6 expression group was lower than that of the high PAX6 expression group, but the difference was not significant (1.19 ± 0.44 vs. 1.38 ± 0.36, *p* = 0.28). Whereas, the low PAX6 expression group had a significantly lower enhancement ratio in the portal phase than the high PAX6 expression group (1.12 ± 0.28 vs. 1.30 ± 0.22, *p* = 0.044). ROC curves based on arterial and portal enhancement ratio and optimal cutoff values for differentiating low PAX6 from high PAX6 Pan-NENs are shown in Figure 7. The optimal cut-off value of the portal enhancement ratio was ≤1.22. This had a sensitivity and specificity of 72.7%, and 70.0%, respectively, and the area under the curve was 0.70 (95% CI: 0.49–0.92).

The sensitivity, specificity, accuracy, positive likelihood ratio, and negative likelihood ratio for significant enhancement features and their combinations are presented in Table 4. The combination of iso- or hypoenhancement types in the arterial phase, iso- or hypoenhancement types in the portal phase, and portal enhancement ratio ≤1.22 had a sensitivity, specificity, accuracy, positive likelihood ratio, and negative likelihood ratio of 54.5%, 92.5%, 84.3%, 7.27, and 0.49, respectively, for predicting low PAX6 Pan-NENs.

### 3.4. Morphological Features and Enhancement Patterns of Pan-NENs Associated with Ki-67 Proliferative Index and Mitotic Count

The morphological features, according to Ki-67 proliferative index and mitotic count, are shown in Table 5. The median tumor size was significantly larger for high Ki-67 and mitotic groups than for low Ki-67 and mitotic groups (28.3 mm vs. 15.0 mm, *p* = 0.012 for the Ki-67 proliferative index; 57.9 mm vs. 15.3 mm, *p* = 0.004 for the mitotic count, respectively). The high Ki-67 group showed ill-defined margins compared with the low Ki-67 group, and this difference was significant (*n* = 5/16, 31.3% vs. *n* = 2/35, 5.7%, *p* = 0.025). Calcifications were observed to occur significantly more frequently in both high Ki-67 and mitotic groups than in low Ki-67 and mitotic groups (*n* = 6/16, 37.5% vs. *n* = 2/35, 5.7%, *p* = 0.008 for Ki-67 proliferative index; *n* = 3/4, 75.0% vs. *n* = 5/47, 10.6%, *p* = 0.009 for mitotic count, respectively). Table 6 shows the enhancement patterns according to the Ki-67 proliferative index and the mitotic count. Iso- or hypoenhancement types in the arterial phase were significantly associated with the high Ki-67 group compared with the low Ki-67 group (*n* = 8/16, 50.0% vs. *n* = 7/35, 20.0%, *p* = 0.046). No other significant differences were observed in the enhancement patterns between the low and high Ki-67 or mitotic groups.

## 4. Discussion

Biological factors, such as protein expression, epigenetics, and gene expression in Pan-NENs are expected to optimize prognostic stratification and help plan personalized treatment [36]. Integrating imaging features and the biological information could increase precision in the diagnosis, assessment of the prognosis, and prediction of the treatment response [37]. PAX6 is a critical factor for the differentiation of the pancreas endocrine cells and necessary to control the specification of each hormone-producing endocrine cells [12,13,14,15]. Recently, we showed the relationship of low PAX6 expression with the occurrence of metachronous metastasis and poor survival [22]. The non-invasive prediction technique of low expression of PAX6 in Pan-NENs is desirable for planning appropriate treatment to improve prognosis. Our results showed that among CT enhancement patterns, iso- or hypo-enhancement types in both the arterial and portal phase and portal enhancement ratio of ≤1.22 was useful in predicting Pan-NENs with low PAX6 expression. The combination of these three features showed high specificity and accuracy. To the best of our knowledge, this is the first study to investigate the association between imaging features in Pan-NENs and PAX6 expression.

In this present study, iso- or hypoenhancement types in both the arterial and portal phases were associated with low PAX6 expression. Moreover, NET G2 and NEC G3 Pan-NENs demonstrated low PAX6 expression more frequently. Previous findings have indicated that high-grade Pan-NENs were correlated with low blood flow in perfusion CT and iso- or hypoenhancement types on dynamic CT [32,33,38,39]. Decreased blood flow in iso- or hypo-enhancement types might result from the downregulation of PAX6 expression.

Portal enhancement ratio was significantly lower in the low than in the high PAX6 expression group. Although its sensitivity and specificity were insufficient, the cut-off value of ≤1.22 showed the best performance for differentiating low PAX6 from high PAX6 expression of Pan-NENs. Kim et al. [40] and Belousova et al. [34] showed that the enhancement ratio in the portal phase was associated with histologic grade of Pan-NENs. The higher-grade Pan-NENs tended to have lower values of enhancement ratio in the portal phase [34,40]. Collectively, these results indicate that a lower enhancement ratio in the portal phase may be a potential indicator for Pan-NENs with low PAX6 expression. The arterial enhancement ratio was lower in the low than in the high PAX6 expression group, although not significant. In contrast, the qualitative evaluation showed a significant association between low and high PAX6 expression groups. Ideally, quantitative evaluation findings should align with qualitative evaluation findings. Previous studies have shown that the arterial enhancement ratio was also associated with the histologic grade of Pan-NENs [34,40]. The present study might be limited in detecting a significant difference in the arterial enhancement ratio because of the small number of patients in the low PAX6 group.

Tumor size and calcification were associated with high Ki-67 and miotic group count, and ill-defined margins were significantly observed in the high Ki-67 group. These morphological features were considered as tumor aggressiveness and several previous studies supported this finding [40,41,42,43]. In terms of enhancement patterns, the main enhancement types in the arterial phase were significantly different according to the Ki-67 proliferative index. Our findings are analogous with those of previous studies that indicated Pan-NENs with higher Ki-67 proliferative index showed low blood flow and appeared as iso- or hypoenhancement types [38,41,44]. Unlike the result of Ki-67 and miotic groups, we could not find a significant association between morphological features and PAX6 expression, whereas significant differences in the enhancement patterns were more observed in the PAX6 expression group than in the Ki-67 or miotic groups. These facts might indicate that PAX6 expression contributes to determining the enhancement patterns of Pan-NENs more than morphological features.

The enhancement patterns on dynamic CT may explain the biological characteristics of PAX6 in Pan-NENs. Typical Pan-NENs preserve high vascularization and high PAX6 expression, as they originate from the pancreatic islet. Our results showed that PAX6 downregulation was associated with impaired angiogenesis in Pan-NENs, resulting in low vascularization. Angiogenesis is regulated by multiple molecular factors such as the vascular endothelial growth factor family, platelet-derived growth factor, and epidermal growth factor [45,46]. However, the association of PAX6 with these factors has not been identified in Pan-NENs. Tang et al. recently showed that PAX6 regulates the function of epidermal growth factor like domain multiple 6 and promotes angiogenesis in small bowel vascular malformation disease [47]. In glioma cells, PAX6 suppressed the expression of vascular endothelial growth factor A and the angiogenesis of glioma cells [48]. Interestingly, PAX6 expression of glioma cells seems to be inversely correlated with angiogenesis in Pan-NENs. Further studies are needed to investigate the association between PAX6 and angiogenesis factors in Pan-NENs and clarify the differences in the association between PAX6 expression and angiogenesis observed in Pan-NENs and other tumors.

In this study, the prediction of low PAX6 expression in Pan-NENs was solely analyzed based on CT features. Currently, various imaging techniques such as CT, magnetic resonance imaging (MRI), and positron emission tomography (PET)/CT play a key role in staging and determining the treatment of Pan-NENs [49]. MRI shows higher soft-tissue contrast resolution compared to CT; thus, it has an advantage for finding the lesions of Pan-NENs [28]. PET/CT is generally useful in identifying the unknown primary or detecting metastatic lesions, which cannot be found by CT or MRI. There are several ^68^Ga-radiolabelled somatostatin analogs that show high sensitivity (>90%) and specificity (92–98%) for finding NETs [50,51]. Conversely, ^18^F-fludeoxyglucose uptake is observed in the poorly differentiated tumors that show the low expression of somatostatin receptors [52,53]. In the future, it is desirable to clarify the relationship between PAX6 expression in Pan-NENs and imaging findings of modalities other than CT. It is then necessary to investigate the most useful imaging technique for predicting low PAX6 expression or whether the combination of multi-modality imaging may improve the prediction of low PAX6 expression in Pan-NENs. 

Our study has some limitations. First, research on the value of PAX6 expression of Pan-NENs in predicting hepatic metastasis or survival remains limited. Further studies are needed to evaluate the value of PAX6 expression in patients with Pan-NENs. Second, our cohort was small, and the number of patients in each PAX6 expression group was uneven. There may be more critical imaging features associated with low expression of PAX6 in Pan-NENs. Generally, Pan-NENs are rare tumors, and PAX6 is demonstrated to be immunohistochemically positive in the majority of Pan-NENs. Therefore, it was challenging to obtain the same number of patients in each group according to PAX6 expression in a retrospective study. Third, some patients who had undergone preoperative dynamic CT studies outside of our institutions (approximately 20%) were included because of the retrospective design of the study, and variance in the CT protocols is a major concern. Further validation studies in large populations and using the unified dynamic CT protocol are needed to confirm the relationship between enhancement types and PAX6 expression in Pan-NENs.

In conclusion, arterial and portal iso- or hypoenhancement types and low portal enhancement ratio on dynamic CT may be predictive factors for Pan-NENs with low PAX6 expression. These predictive factors may enable us to identify poor outcomes of Pan-NENs patients with no synchronous liver metastasis and help in planning appropriate treatment to improve prognosis.

## Figures and Tables

**Table 1:** Patient characteristics according to PAX6 expression.

Characteristic	Low PAX6 Expression Group (*n* = 11)	High PAX6 Expression Group (*n* = 40)	*p* Value ^a^
Age (y) ^b^	53 ± 15	61 ± 11	0.14
Male sex	6	16	0.50
Genetic disorder	1	1	0.39
Time from CT to surgery (d) ^c^	24 [6.5, 43]	34 [8.75, 76.5]	0.43
2010 WHO classification			
NET G1	6	33	0.049
NET G2	4	7	
NEC G3	1	0	
Ki-67 index (%) ^b^	6.25 ± 8.85	1.81 ± 1.72	0.021
Mitotic count (n/10 HPF) ^b^	1.45 ± 1.86	0.80 ± 0.94	0.11
Symptomatic tumor	2	13	0.47
Metachronous hepatic metastasis	2	1	0.11
Death	2	0	0.043

Unless otherwise specified, data indicate the number of patients. ^a^ *p* values were calculated using Fisher’s exact test for categorical variables and Mann–Whitney U test for continuous variables. ^b^ mean ± standard deviation. ^c^ median [1st quartile, 3rd quartile]. Abbreviations: PAX6, paired box 6; CT, computed tomography; NET G1, neuroendocrine tumor grade 1; NET G2, neuroendocrine tumor grade 2; NEC G3, neuroendocrine carcinoma grade 3; HPF, high power field.

**Table 2:** Morphological features according to PAX6 expression.

Features	Low PAX6 Expression Group (*n* = 11)	High PAX6 Expression Group (*n* = 40)	k	*p* Value ^a^
Size (mm) ^b^	18.6 [13.5, 44.0]	15.5 [10.1, 21.8]		0.24
Location				
Head	5	16	0.76	0.28
Body	0	9		
Tail	6	15		
Shape				
Round	2	15	0.50	0.44
Oval	7	21		
Lobulated	2	4		
Margin (well-defined)	9	35	0.45	0.63
Cystic change	1	5	0.46	1.00
Calcification	4	4	0.92	0.055
Visible intratumoral vessel	1	5	0.73	1.00
Invasion into adjacent vascular	2	3	0.63	0.29
Main pancreatic duct dilatation	1	2	0.84	0.52
Upstream pancreatic atrophy	2	1	0.64	0.11

Unless otherwise specified, data indicate the number of patients. ^a^ *p* values were calculated using Fisher’s exact test for categorical variables and Mann–Whitney U test for continuous variables. ^b^ median [1st quartile, 3rd quartile]. Abbreviation: PAX6, paired box 6.

**Table 3:** Enhancement patterns according to PAX6 expression.

	Low PAX6 Expression Group (*n* = 11)	High PAX6 Expression Group (*n* = 40)	k	*p* Value ^a^
Tumor homogeneity				
Arterial phase				
Homogeneous	8	26	0.69	0.73
Heterogeneous	3	14		
Portal phase				
Homogeneous	8	29	0.77	1.00
Heterogeneous	3	11		
Main enhancement types				
Arterial phase				
Hyperenhancement	4	32	0.64	0.009
Iso- or hypoenhancement ^b^	7 (3, 4)	8 (6, 2)		
Portal phase				
Hyperenhancement	3	33	0.76	0.001
Iso- or hypoenhancement ^b^	8 (6, 2)	7 (6, 1)		
Enhancement ratio ^c^				
Arterial phase	1.19 ± 0.44	1.38 ± 0.36		0.28
Portal phase	1.12 ± 0.28	1.30 ± 0.22		0.044

Unless otherwise specified, data indicate the number of patients. ^a^ *p* values were calculated using Fisher’s exact test for categorical variables and Mann–Whitney U test for continuous variables. ^b^ Data in parentheses are number of iso- and hypoenhancement tumors. ^c^ mean ± standard deviation. Abbreviation: PAX6, paired box 6.

**Table 4:** Diagnostic performance of the three significant enhancement features and their combination in predicting low PAX6 Pan-NENs.

Parameter	Sensitivity (%)	Specificity (%)	Accuracy (%)	Positive LR	Negative LR
Iso- or hypoenhancement types on the arterial phase	63.6 (30.8, 89.1) [7/11]	80.0 (64.4, 90.9) [32/40]	76.5 (62.5, 87.2) [39/51]	3.18 (1.48, 6.83)	0.46 (0.21, 1.01)
Iso- or hypoenhancement types on the portal phase	72.7 (39.0, 94.0) [8/11]	82.5 (67.2, 92.7) [33/40]	80.4 (66.9, 90.2) [41/51]	4.16 (1.94, 8.92)	0.33 (0.13, 0.88)
Enhancement ratio on the portal phase ≤ 1.22	72.7 (39.0, 94.0) [8/11]	70.0 (53.5, 83.4) [28/40]	70.6 (56.2, 82.5) [36/51]	2.42 (1.34, 4.40)	0.39 (0.15, 1.04)
At least one parameter	81.8 (48.2, 97.7) [9/11]	60.0 (43.3, 75.1) [24/40]	64.7 (50.1, 77.6) [33/51]	2.05 (1.28, 3.26)	0.30 (0.08, 1.09)
Any two parameters	72.7 (39.0, 94.0) [8/11]	80.0 (64.4, 90.9) [32/40]	78.4 (64.7, 88.7) [40/51]	3.64 (1.77, 7.45)	0.34 (0.13, 0.91)
All three parameters	54.5 (23.4, 83.3) [6/11]	92.5 (79.6, 98.4) [37/40]	84.3 (71.4, 93.0) [43/51]	7.27 (2.16, 24.5)	0.49 (0.26, 0.94)

Data in parentheses are 95% confidence intervals; data in brackets are numerator/denominator. Abbreviation: LR, likelihood ratio.

**Table 5:** Morphological features according to Ki-67 proliferative index and mitotic count.

	Ki-67 Proliferative Index			Mitotic Count		
Features	≤2%: Low Group (*n* = 35)	>2%: High Group (*n* = 16)	*p* Value ^a^	<2 *n*/10 HPF: Low Group (*n* = 47)	≥2 *n*/10 HPF: High Group (*n* = 4)	*p* Value ^a^
Size (mm) ^b^	15.0 [10.6, 17.8]	28.3 [14.5, 54.3]	0.012	15.3 [10.0, 20.1]	57.9 [42.2, 76.7]	0.004
Location						
Head	14	7	0.85	19	2	1.00
Body	7	2		9	0	
Tail	14	7		19	2	
Shape						
Round	13	4	0.15	17	0	0.24
Oval	20	8		25	3	
Lobulated	2	4		5	1	
Margin (well-defined)	33	11	0.025	41	3	0.46
Cystic change	3	3	0.36	4	2	0.063
Calcification	2	6	0.008	5	3	0.009
Visible intratumoral vessel	2	4	0.069	5	1	0.40
Invasion into adjacent vascular	2	3	0.309	4	1	0.35
Main pancreatic duct dilatation	1	2	0.23	2	1	0.22
Upstream pancreatic atrophy	1	2	0.23	2	1	0.22

Unless otherwise specified, data indicate the number of patients. ^a^ *p* values were calculated using Fisher’s exact test for categorical variables and Mann–Whitney U test for continuous variables. ^b^ median [1st quartile, 3rd quartile]. Abbreviation: HPF, high power field.

**Table 6:** Enhancement patterns according to Ki-67 proliferative index and mitotic count.

	Ki-67 Proliferative Index			Mitotic Count		
Features	≤2%: Low Group (*n* = 35)	>2%: High Group (*n* = 16)	*p* Value ^a^	<2 *n*/10 HPF: Low Group (*n* = 47)	≥2 *n*/10 HPF: High Group (*n* = 4)	*p* Value ^a^
Tumor homogeneity						
Arterial phase						
Homogeneous	24	10	0.75	33	1	0.10
Heterogeneous	11	6		14	3	
Portal phase						
Homogeneous	27	10	0.32	36	1	0.057
Heterogeneous	8	6		11	3	
Main enhancement types						
Arterial phase						
Hyperenhancement	28	8	0.046	34	2	0.57
Iso- or hypoenhancement ^b^	7 (3, 4)	8 (6, 2)		13 (8, 5)	2 (1, 1)	
Portal phase						
Hyperenhancement	25	11	1.00	33	3	1.00
Iso- or hypoenhancement ^b^	10 (8, 2)	5 (4, 1)		14 (12, 2)	1 (0, 1)	
Enhancement ratio ^c^						
Arterial phase	1.38 ± 0.41	1.24 ± 0.32	0.096	1.36 ± 0.39	1.13 ± 0.20	0.19
Portal phase	1.27 ± 0.23	1.27 ± 0.27	0.96	1.26 ± 0.23	1.24 ± 0.39	0.57

Unless otherwise specified, data indicate the number of patients. ^a^ *p* values were calculated using Fisher’s exact test for categorical variables and Mann–Whitney U test for continuous variables. ^b^ Data in parentheses are the number of iso- and hypoenhancement tumors, respectively. ^c^ mean ± standard deviation. Abbreviation: HPF, high power field.

## Appendix A

### A.1. Injection protocol of CT for our institution

After unenhanced scans were acquired, the contrast media with iodine concentrations of 300 mg/mL were intravenously administered, amounting to 2.0 mL/kg of body weight, at a rate of 3 mL/s using a pump injector. The scanning timings for GE HiSpeed CT (GE Healthcare, Chicago, U.S.A.) were as follows: The arterial and portal phase scans were obtained at 30 and 70 s after injecting the contrast media. The scanning timings for Aquilion 64-slice CT (Canon medical systems, Tochigi, Japan) were as follows: the arterial phase scan was obtained at 15 s after aortic attenuation reached 150 Hounsfield units (HUs). The portal phase scans were obtained at 80–100 s after injecting the contrast media. Thus, all these CT studies included unenhanced scans and the arterial and portal phase scans.
